# Mental health problems in Pakistani society as a consequence of violence and trauma: a case for better integration of care

**DOI:** 10.5334/ijic.662

**Published:** 2011-10-07

**Authors:** Muhammad Tahir Khalily

**Affiliations:** Psychology Department, Roscommon Mental Health Services; Clinical Supervisor for D.Psych.Sc, School of Psychology National University of Ireland, Galway, Ireland

**Keywords:** mental health, primary care, Pakistan, health policy, conflict, post-traumatic stress disorder

## Abstract

**Objectives:**

This paper discusses the increasing incidence of mental health problems in Pakistan, and specifically in the Swat Valley, in relation to the growing insurgency and current violence in Pakistani society. The paper argues that the health care system’s response in Pakistan is not adequate to meet the current challenges and that changes in policy are needed to build mental health care services as an important component of the basic health package at primary care level in the public sector.

**Method:**

This paper reviews the existing mental health situation in Pakistan with reference to the findings of a case study in the Swat Valley in Khyber Pukhtoonkhwa Pakistan. The figures presented in the case study are used to support the need for an integrated national mental health policy.

**Conclusion:**

Mental health care needs to be incorporated as a core service in primary care and supported by specialist services. There is a strong need to provide adequate training for general practitioners and postgraduate training for mental health professionals to meet the current demands. A collaborative network between stakeholders in the public and private sector, as well as non-governmental organisations are required that promotes mental health care and advocates for changes in mental health policy.

## Introduction

There is an alarming increase in the incidence of mental illness due to a persistent wave of violence, political turmoil and frequent changes in the social fabric in many countries worldwide [1, 2]. As a result of this, a range of psychiatric disorders have been reported, such as depression, substance and alcohol misuse, schizophrenia, bipolar disorder, and post-traumatic stress disorder [3]. More suicides are committed every year in both developed and developing countries as a result of mental health problems [3].

Mental health problems in Pakistan, a developing country, have in the last few decades reached an appalling level [4, 5] linked to both the current violence in Pakistani society [6, 7] and disruption in its social structure [8]. Many researchers are agreed that the psychological outcome of the communities as a whole will be that of resilience rather than psychopathology [9]. Nevertheless, a greater magnitude of exposure to traumatic events is known to be associated with the greater prevalence of severe mental health problems [10]. This continuous violence and threat to life has had a damaging effect to the psychological health of many people in general [11] and particularly in Pakistani society [6].

The health care treatment system’s response to these problems is different in developed and developing countries across almost all dimensions including: policy, the role of educational institutions, financial resources, infrastructural development, public-private partnership, academic and research endeavours, civic amenities and human rights issues [3]. Developed countries are further ahead in utilizing more resources and having consistent comprehensive educational and treatment policies to deal with mental health issues effectively [12]. In developing countries, the number of psychiatrists and psychiatric beds per head of population is much smaller and the majority of people having psychiatric disorders cannot afford the treatment expenses since these must usually come from their own pocket and where there is low average annual income. Fundamentally, there is no established model for mental health care in most developing countries and the majority of psychiatric patients thus seek treatment from non-professional healers using psychobabble—psychological jargon used inaccurately to talk about someone’s personal or mental problems. In developed countries, by contrast, the availability of community-based psychiatric services led by trained professionals and supported by specialists is the established treatment system [3]. Access to such services rarely comes with an out-of-pocket payment since taxation or insurance-based funding is used.

## Current mental health issues in Pakistan

The prevalence of mental health problems in Pakistan is increasing rapidly due to current violent situation in Pakistani society [5, 7]. Common mental health problems have been identified in both the rural and urban population [4] which seems to have a positive association with socio-economic adversities, relationship problems and lack of social support [13]. Depressive and anxiety disorders appear to be highest [4, 14] followed by bipolar, schizophrenia, psychosomatic disorders, obsessive compulsive disorder [3] and post-traumatic stress disorder [6]. There is also a high prevalence of depression amongst Afghan refugees residing in Pakistan [14]. In addition, there is a serious problem of substance misuse and drug addiction. About four million drug addicts have been estimated in the last national survey in Pakistan [15] with a growing number of injectable drug users in the urban population creating the public health predicament [16]. However, the incidence among adults is under-reported due to social stigma in the context of family pride [17]. Nevertheless, the current wave of violence and aggression in Pakistani society is not a simple phenomenon [18]. There has been an increase in violence over the past five years in Pakistan, such as suicide attacks, explosions, and even safety precautions, such as long curfew hours have caused damage on an unprecedented scale [6]. The local inhabitants have experienced a heavy battle between the security forces and insurgents. In addition to the continuous violence and threat to life, there has also been a damaging effect to the psychological health of many people [19]. Psychological trauma as consequence of violence is on the rise, prevailing in the whole area [18]. As a result, individuals are manifesting a number of symptoms of psychological trauma, which is affecting all aspects of their lives.

## Post-traumatic stress disorder in the Swat Valley

To illustrate the depths of these problems, the next section of this paper examines evidence collected on the prevalence of post-traumatic stress disorder (PTSD) amongst young people in the Swat Valley of the Khyber Pukhtoonkhwa, the fourth province in the North West part of Pakistan, where arguably the greatest levels of violence and insurgency are to be found. In particular, the paper presents the results of a survey of (n=600 participants) young people (age range 11–22 years) conducted by the Department of Community Health Sciences at Hamdard College of Medicine and Dentistry Karachi [20]. The findings are used to build a case for a policy change to support integrating mental health into local primary care services.

**Figure 1:**
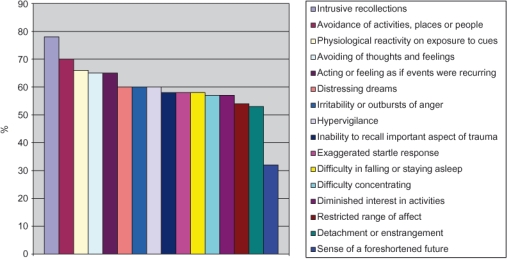
Presence of PTSD symptoms in a population of (n=600), of age range 11–22 years in the Swat Valley.

**Figure 2:**
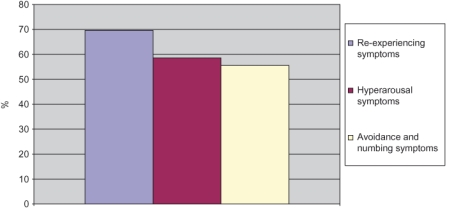
PTSD symptoms in clusters in a population of (n=600) age range 11–22 years in the Swat Valley.

The result of this study are provided in Figures 1 and 2. Figure 1 shows that the subjects had experienced 16 out of 17 symptoms of PTSD (as set out in criteria mentioned in the Diagnostic and statistical Manual of Mental Disorders-1V (DSM-1V) [21]. This ranged from a high of 79% for ‘psychological distress and exposure to cues’ to a low of 32% for ‘sense of a foreshortened future’ which in the context of the results indicates their pragmatic view about the future, which is a more positive indicator of their resilience. However, it appears from the evidence that some people will be more affected by a traumatic event for a longer period of time than others, depending on their gender, the nature of the event, such as devastation and destruction that has occurred, as well as injuries and lives lost, and the individual who experienced the event [10].

Figure 2 portrays PTSD symptoms in three clusters, such as re-experiencing symptoms (69.6%), avoidance and numbing symptoms (58.6%) and hyper-arousal symptoms (55.8%). These results establish the presence of PTSD symptoms both individually and in clusters in this sample as a result of exposure to extreme traumatic stressor as often seen in the trauma survivors [6, 25].

To put these results into context, similar studies have been undertaken in Afghanistan [22], Sri Lanka [23], and Algeria [24] in conflict situations. In each case, the studies sought to establish the potential link of the aftermath of conflict to psychological issues and higher rates of prevalence of post-traumatic stress disorder (PTSD) among the survivors. From these studies, the possibility of developing PTSD can be seen to be a function of many variables, the most important being exposure to traumatic events. PTSD is a highly widespread lifetime disorder that frequently continues for years [25], with an increasing recognition of deep and long-lasting detrimental effects on health status and quality of life [26]. Recent studies have suggested that a significant proportion of the population may experience delayed PTSD symptoms, whereby individuals exposed to a traumatic event do not meet criteria at an initial stage, but do meet criteria at a later point [27, 28]. In addition, PTSD has a direct association with substance abuse, self-harm and other co-morbid psychiatric disorders [29], which are already known to be major mental health issues in Pakistani society [6, 7]. Subsequently, deterioration in mental health is a negative indicator for the psychological, physical, social and economic development of a society, and a poor socio-economic status is closely linked to mental illness in general and to substance abuse in particular [4, 16, 30]. So it is pertinent to have a vigilant mental health care system to deal with these issues in time and effectively.

In the next section an attempt has been made to review the present treatment services and to propose a strategy compatible with Pakistan’s organizational culture and infrastructure to suggest changes at the policy level, to improve the existing facilities of mental health treatment in line with international standards, which would ultimately play a significant role in the mental health promotion and development of our society in general.

## Mental health and health care system response

The health care system’s response, particularly the mental health care in the Swat Valley, is not keeping pace with the growing incidence of mental illness. The mental health services are still under-resourced in terms of trained professionals [31], community care for patients [3] and meagre financial resources (mostly limited to the cities in spite of the fact that the majority of the population resides in the countryside) [32]. Even so, available facilities are under-utilized as a result of the social stigma associated with psychiatric labelling [12] and a popular misconception in the community that mental illnesses are due to the possession of “Jin” or evil eyes or “Jadho” (magic) seemingly confirmed when patients consult traditional healers whose caseloads are often dominated by mental disorders [23].

There are few mental health professionals, including psychiatrists, psychologists and social workers, to provide mental health treatment in the country [7]. In the Swat Valley, there is only one psychiatrist for more than a population of two million with no allied health professionals. The number of psychiatric beds is smaller in general and particularly in the Swat valley hospital compared to the population [3]. Most people with mental illnesses therefore have no access to psychiatric services of which they are unaware. Either they turn to traditional healers or they live with their disabling psychiatric disorders [33].

Progress in mental health care lags behind that for other medical disciplines and undermined at policy level [34]. Collaboration between medical practitioners and the mental health sector is poorer than in other fields [31]. Teaching psychology in colleges (mostly for boys) is not a part of the curriculum. Teaching the behavioural sciences in the medical schools is not being taken seriously and there is no structural rotation programme for senior medical students who have a low interest in the subject of psychiatry [35]. There is some clinical psychology training centres/departments in the Khyber Pukhtoonkhwa conducting one- or two-year courses. However, the majority of these institutions emphasise teaching rather than clinical supervision and have no clinical placement schedule [36].

### The role of non-governmental organizations

Non-governmental organizations (NGOs) working for the promotion of mental health in Pakistan have been evolving in recent decades, but have not kept pace with the demand for more and better services. Their role is, in any case, limited to the sporadic public awareness programmes, gender discrimination issues, social and cultural activities. They have no comprehensive strategy for the promotion of mental health issues particularly in the Swat Valley. Despite these limitations, these organizations could take the lead role in reviewing mental health policy, updating treatment facilities and moving from institutionalized to community-based psychiatric services less encumbered by the need to go through bureaucratic channels. Moreover, the NGOs could play a significant role in the promotion of mental health policy applying the principles of community psychiatry within geographically defined populations to deliver treatment at the door step by multi-disciplinary teams offering continuity of care and encouraging consumer participation. That approach might well be built into public private partnership [31] integrated into the basic health package as proposed in the next section.

### Integration of mental health in primary care

The mental health situation in Pakistan demands pressing attention from policy makers, professional bodies, academics and professionals working in the mental health field [37] to review the existing policies and to work for an integrated national mental health policy (as suggested in Table 1) [38].

Utilization of existing facilities would be vital to minimize capital expenditure and to make the psychiatric services cost effective. A primary care (Basic Health Unit (BHU)/Rural Health Centre) and secondary care system (District Head Quarter Hospitals (DHQ) and Teaching Hospitals) are already in place in the health care system of Pakistan including the Swat Valley.

BHU/RHC→DHQ/Teaching Hospitals

Adequate training in psychiatry for a mental health professional and for general medical practitioners in the primary care units can bring significant improvement in the mental health care in terms of early diagnosis (as late presentation damages the prognosis of people with schizophrenia), appropriate treatment and referral to a specialist psychiatric service [39, 40]. The professional bodies, particularly the Pakistan Association for Mental Health and Pakistan Association for Clinical Psychologists can lead in collaboration with other organizations to work for a national mental health policy as proposed (Table 1). There is, however, no academic, professional or third-level course in Pakistan at university level to study mental health problems profoundly and in line with international standards [37]. A centre of excellence is needed to provide diploma, masters and doctorate-level courses to study mental health (with an emphasis on psychological trauma, addiction and neuropsychological disorders) in-depth as a major public health issue. Postgraduate training in psychiatry and clinical psychology may be encouraged among female mental health professionals keeping in view social and cultural values of this region [41, 42]. Movement for global mental health [43] and the role of media would be significant in promoting mental health [44].

## Conclusion

Mental illnesses have reached an appalling level in Pakistan, particularly in Swat Valley, due to the rising insecurity and persistent violence for many years. The health care system’s response to mental health issues is not compatible as the mental illnesses exacerbates. There is a pressing need to review the national health policy to integrate a mental health care system to primary care to ameliorate the situation. The government needs to be persuaded to adopt a strategic plan integrated within its national development and health strategies. The collaboration of the public health sector, professional bodies in the mental health and the non-governmental organizations is vital to deal with the current challenges. A post-graduate training in mental health is recommended.

## Limitations

1. There is no national epidemiological study available to provide an overall situation of the mental health problems in Pakistan.
2. The insurgency is still emerging from time to time in different parts of the country at the time of writing this paper, the incidence of mental illnesses could be exacerbated, which needs a comprehensive national survey and leave room for policy revision in future.

## Figures and Tables

**Table 1.**
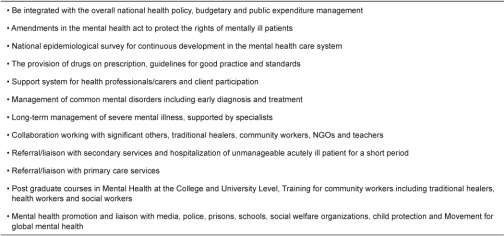
Important features of integrated national mental health policy
